# Understanding action control of daily walking behavior among dog owners: a community survey

**DOI:** 10.1186/s12889-016-3814-2

**Published:** 2016-11-16

**Authors:** Ryan E. Rhodes, Clarise Lim

**Affiliations:** Behavioural Medicine Laboratory, School of Exercise Science, Physical and Health Education, University of Victoria, PO Box 3010 STN CSC, Victoria, B.C. V8W 3N4 Canada

**Keywords:** Identity, Planning, Habit, Affective Attitude

## Abstract

**Background:**

Walking among dog owners may be a means to achieve health benefits, yet almost half of owners (approximately 30% of households) are not regularly walking their dogs. Current research on the correlates of dog walking has generally considered intention as the primary determinant of behavior, yet the intention-behavior relationship is modest. The purpose of this paper was to apply a framework designed to evaluate the intention-behavior gap, known as multi-process action control (M-PAC), to understand daily walking among dog owners.

**Method:**

A community sample of adult dog owners (*N* = 227) in Victoria, Canada completed M-PAC measures of motivational (dog and human outcome expectations, affective judgments, perceived capability and opportunity), regulatory (planning), and reflexive (automaticity, identity) processes as well as intention to walk and behavior.

**Results:**

Three intention-behavior profiles emerged: a) non-intenders who were not active (26%; *n* = 59), b) unsuccessful intenders who failed to enact their positive intentions (33%; *n* = 75), and c) successful intenders who were active (40%; *n* = 91). Congruent with M-PAC, a discriminant function analysis showed that affective judgements (*r* = 0.33), automaticity (*r* = 0.38), and planning (*r* = 0.33) distinguished between all three intention-behavior profiles, while identity (*r* = 0.22) and dog breed size (*r* = 0.28) differentiated between successful and unsuccessful intenders.

**Conclusions:**

The majority of dog owners have positive intentions to walk, yet almost half fail to meet these intentions. Interventions focused on affective judgments (e.g., more enjoyable places to walk), behavioral regulation (e.g., setting a concrete plan), habit (e.g., making routines and cues) and identity formation (e.g., affirmations of commitment) may help overcome difficulties with translating these intentions into action, thus increasing overall levels of walking.

## Background

Regular physical activity (PA) among adults has an enormous number of health benefits [1], but very low participation rates (e.g., [2]). Consequently, PA promotion initiatives are of high importance to public health. Regular walking is one of the most preferred PAs and thus a key target for intervention [3]. One correlate of regular walking that has seen considerable attention in public health is dog ownership. Specifically, dog owners report more walking during leisure-time than non-owners [4–6]. While this is an interesting descriptive finding, its direct application to PA promotion is less practical, as dog ownership is an enormous responsibility with cost implications. Still, approximately 30% of the population in developed countries own dogs [7], and it is estimated that half of all dog owners do not walk their dogs regularly [4]. While dog walking is merely one of many types of PAs that dog owners could potentially enact, it seems a logical way to engender both human and canine health benefits simultaneously among this large potential target population [8, 9]. Understanding the correlates of walking would thus help identify key intervention targets to promote owners to walk their dogs more.

A recent review of 31 studies on the correlates of walking among dog owners found that an attachment to the dog in the form of responsibility/obligation/support and environmental access to suitable walking areas with dog supportive features (e.g., off-leash exercise) were reliable factors [10]. More recently, Richards et al. [11] have shown that social cognitive theory constructs of dog outcome expectations, social support and measures of the walking environment were key predictors of regular walking. Other studies have also shown some evidence that the theory of planned behavior can predict regular dog walking [12, 13]. Still, the Westgarth et al. review noted that only a handful of the studies used a theoretical framework to explain walking and this is a noteworthy limitation.

An additional potential limitation to prior work is the positioning of intention as the proximal determinant of walking behavior. Indeed, even Westgarth and colleagues [10] suggested that all correlates of dog walking should affect behavior through intention. This positioning makes the assumption that once a dog owner has positive intentions to walk, it will be sufficient to enact the behavior. Still, there is only modest support for the relationship between intention and behavior in general PA research [14]. Interestingly, the relationship between intention and behavior also shows that almost all discordance occurs from those who intend but fail to perform the behavior, and not from those with low intentions who enact the behavior [14]. The sizeable proportion of intenders who subsequently fail to follow-through and enact behavior has prompted the term ‘intention-behavior gap’, because approaches that feature intention as the proximal determinant of behavior have limited theoretical explanation for this finding [15]. It seems a worthy line of inquiry to examine the intention-behavior gap in dog owners’ dog walking behavior and this has not been formally explored at present.

Several models have attempted to understand the translation of intention into behavior, also known as action control [16]. One of the most frequently applied of these in the PA domain is the multi-process action control framework (M-PAC; [16–19]). In this framework, intention (i.e., intend/do not intend) and behavior are divided into quadrants, which creates four possible profiles, but only three of substantive value: *non-intenders* who are subsequently not active; *successful intenders* who are subsequently active, and *unsuccessful intenders* who failed to enact their positive intentions. By contrast, the fourth profile of *disinclined actors,* who despite lack of intention are subsequently active, is hypothesized as empty because intention is viewed as a necessary but insufficient process to achieve PA within M-PAC [16, 17].

The intention-behavior profiles in M-PAC have similarities to the stages of change in the transtheoretical model [20] as intention-behavior hybrid constructs, but M-PAC profiles are not stages, as someone can move from non-intender to successful intender without ever falling into the “unsuccessful intender” profile. M-PAC suggests that intenders may be predicted by motivational processes of instrumental attitude/outcome expectations (utility of the behavior), affective judgments (enjoyment of the behavior) and perceived control (ability and opportunity to perform the behavior) which supports the tenets of most intention-based theories [21]. In M-PAC, however, affective judgments and opportunity are also considered predictors during the intention-formation to action control process, where higher values are considered necessary for successful translation of intentions into behavior than for intention formation. Furthermore, action control is thought to be dependent on regulation behaviors (e.g., planning, self-monitoring), as people begin to use volitional tactics to help translate positive intentions into action. Regulation behaviors are conceptually similar to action/coping planning in the health action process approach [22] or the behavioral processes of change in the transtheoretical model [20].

Continuance of action control is thought to also add reflexive processes such as automaticity/habit (i.e., behavior performed from stimulus–response bonds) and identity (self-categorisation) as one begins to perform the behavior more regularly. Specifically, as a behavior has become more routine, Rhodes and de Bruijn [17] suggest that intention-driven behavior is executed partially from environmental cues [23], selective processing of information congruent with one’s self-categorization [24] and the dissonance that arises from any discrepancy between self-categorization and behavior [25]. To date, M-PAC has not been applied to understand dog walking behavior but it may show utility, given the likelihood of the intention-behavior gap. Furthermore, the regulatory and reflexive processes in M-PAC offer constructs that have not yet been examined within this domain. As much of this walking behavior has the potential to be ritualized and routine, habit also seems like a worthy concept in this domain. Further, as dog-owner attachment constructs have prior validation with owner dog walking [10], an exploration of dog-walking identity in action control may be important.

Thus, the purpose of this paper was to apply the M-PAC framework in a sample of dog owners to understand the translation of daily walking intention and behavior. It was hypothesized that participants would group into three (i.e., non-intenders, unsuccessful intenders, successful intenders) of the four possible intention-behavior profiles based on prior research in general physical activity contexts [14]. It was further hypothesized that affective judgments (enjoyment of owner dog walking), opportunity (availability of time and environment to walk), regulation behaviors (detailed plans to dog walk) and reflexive processes of habit (learned responses to walking cues) and identity (personal standards of dog walking behavior) would explain successful, compared to unsuccessful, intenders based on prior research with this model [17].

## Methods

### Study design

This study featured a cross-sectional survey of adults who were dog owners in the Greater Victoria, Canada region.

### Participants and recruitment

The details of the sample and the recruitment methods have been reported elsewhere [26], although this present study does not contain any of the same measures used from this prior report or address similar research questions. Eligibility to participate was delimited to English-speaking adults, 18+ years of age, who lived in Greater Victoria, Canada, and owned at least one healthy dog between one to seven years of age. Owners of young dogs under one year of age and senior dogs were excluded due to the larger variability in health status and physical ability to walk regularly [27]. Following ethical approval, participants were given details of the study and asked for their informed consent online before proceeding to answer the questionnaire. The survey was published online for public access via Fluid Surveys between December 2013 and January 2014. The published link to the survey was shared primarily on various Facebook pages that were involved in canine rescue/rehoming, pet-related services, dog training services, etc. within the Greater Victoria, Canada region. The online survey link was also published on several websites (e.g., researcher’s laboratory website, graduate student society) and disseminated via the graduate student society’s weekly electronic mailing list. Pamphlets containing the study details, online survey link, and researcher’s contact information were also used in reaching out to more people in-person at dog parks; physical posters containing the same information were put up on notice boards located within recreation centres, university campus, libraries, and selected veterinary clinics that agreed to allow recruitment materials to be displayed. Due to the anonymity of the survey, specific technical settings were created to reduce the chances of multiple responses from the same respondent; access to the survey was limited to one time per computer. For every completed response that matched the eligibility criteria, one dollar in Canadian currency was donated to a local dog rescue of the respondent’s choice.

### Instrumentation

*Owner Dog Walking Behavior* was measured using an adapted version of the Godin Leisure-Time Exercise Questionnaire [28] that has been applied in prior related research [13, 29, 30]. Participants were asked to recall their average weekly walking with their dog during their free time over the past week. The questions were phrased to focus on their walking and not the physical activity of their dog during an outing (e.g., off leash running time). The measure contained three open-ended questions asking for the average frequency at 20+ minutes duration of mild (i.e., minimal effort, no perspiration, a casual walk), moderate (i.e., not exhausting, light perspiration, a good brisk pace) and strenuous (i.e., heart beats rapidly, sweating, as fast as you could walk) walking during the past week. The 20+ minutes duration was modified from the original 15+ min Godin Leisure-Time Questionnaire to correspond with the American College of Sports Medicine’s [31] recommendation of 20+ minutes of strenuous physical activity for public health and to match the minimum walking recommendations for canine health [9]. Commensurate with recommended physical activity guidelines for adults [32], we retained and aggregated the moderate and strenuous intensity categories (20+ min bouts) for analyses.

Constructs used in the M-PAC model assessment were framed for daily dog owner walking at least at a moderate intensity (see also Table 3 in Appendix).

*Intention* was measured with the item recommended by Courneya [33] for open-scaled assessment of weekly frequency of PA. The item was phrased “Over the next week, I intend to walk the dog ___________ days per week”.

We measured *human outcome expectations* based on the following two items from prior research [34]: 1) Regular dog walking would help me to maintain or lose weight; 2) Regular dog walking would allow me to get to know my neighbourhood (α = 0.69) and measured *canine-based outcome expectations* with the item: Regular dog walking would help keep my dog healthy*.* The items began with the phrase “Assuming you walked with your dog daily, how likely or unlikely is it that each of the following would occur?” and were scored from 1 (very unlikely) to 5 (very likely).

*Affective Judgments* were measured using the intrinsic regulation scale from the Behavioral Regulations in Exercise Scale-2 [35], adapted for daily dog walking (α = 0.92). Example items included “I *walk my dog* because it is fun” and “I find *dog walking* a pleasurable activity”.

Perceived control over dog walking in the form of *capability* and *opportunity* were measured with items from Rhodes and colleagues [36, 37]. The items were: I am physically able to walk my dog regularly if I wanted to (capability) and I have the opportunity to walk my dog regularly if I wanted to (opportunity), scored from 1 (strongly disagree) to 5 (strongly agree).

*Behavioral regulation* was measured using three items adapted from Sniehotta and colleagues [38] for daily dog walking*.* The measures were scored from strongly disagree (1) to strongly agree (5). The reliability of these three items had a Cronbach’s α. = 0.76. Examples of these items included “I made detailed plans regarding what to do if something interfered with my plans to engage in dog walking over the past week” and “I made plans concerning “when”, “where”, “what” and “how” I was going to engage in regular dog walking over the past week”.

*Habit/Automaticity* was measured with the self-reported automaticity subscale [39]. The measure was scored on a five-point scale from strongly disagree (1) to strongly agree (5) and reliability was satisfactory (α = 0.93). Example items included “I engage in dog walking without consciously thinking about it” and “I engage in dog walking automatically”.

Identity was measured using the three-item exercise role identity subscale [40] adapted for dog walking. Example items included “I consider myself someone who is physically active with my dog” and “When I describe myself to others, I usually include being physically active with my dog*”.* The measure was scored on a five-point scale from strongly disagree (1) to strongly agree (5) and reliability was satisfactory (α = 0.87).

### Analysis Plan

Our M-PAC measures were defined as daily dog walking because this target corresponds well to a mix of both human (i.e., 7 x 20+ min = 140+ min MVPA) and canine (at least daily walking 20+ for all healthy breeds of adult dogs) walking recommendations [9, 41]. Similarly, the creation of the action control framework of intention-behavior profiles was built to correspond with this definition. This allows for a scale correspondent prediction model as both the M-PAC predictors and the action control outcome variable are defined with the same target value of daily dog walking. Using this criterion, intention/behavior scores were dichotomized as below daily walking (<7 days per week) and daily walking (7 days per week). The categorization provided four possible quadrants of: a) non-intenders (low intention, low walking), b) non-intenders who were walking their dogs daily (low intention, high walking), c) unsuccessful intenders (high intention, low walking), and d) successful intenders (high intention, high walking) who were walking at least 140 min per week. To define our minimum cell size needed among these four possible action control categories, we used basic one-way analysis of variance power estimation. Considering a small medium effect size (*f* = .25), an alpha of .05, and a power of .80, 45 participants were needed in a particular intention-behavior profile to be included in the analyses [42]. Based on prior research [14], we expected that participants classified as non-intenders who were walking their dogs daily would not be a large enough group to be included in subsequent analyses but we expected all other groups to be above this *n* = 45 criterion.

Before conducting the multivariate prediction of the action control framework, we examined potential demographic (age, gender, income, occupational status, education), home environment (presence of a back yard), and dog (breed size) characteristics as covariates using analysis of variance and chi-square analyses. Significant covariates (*p* < 0.05) were carried forward to the main analyses. For the main analyses, prediction of action control category membership used discriminant function analysis. Associations with a significant discriminant function used *r* = 0.20 as the minimum recommended effect size for the social sciences based on recent recommendations [43]. For predictors that had a meaningful correlation with the discriminant function, follow-up univariate *F*-tests and Tukey post-hoc difference tests were conducted similar to prior work with the action control framework [17]. Significance was set at *p* < 0.05, but effect sizes were used to aid in the interpretation of the inferential statistics results. Specifically, we used *d* = 0.30 as the minimum recommended effect size because the estimate is between Ferguson’s [43] *d* = 0.41 and Cohen’s [44] *d* = 0.20 recommendations. Our a priori power analysis of this prediction equation suggested that we required a sample size of *N* = 166, considering our estimated small effect size, p-level (0.05), an estimated power of 0.80, and the seven M-PAC predictors with an estimated two additional covariates.

The advantage of the action control framework approach is that researchers can gain insight into intention translation at a particular value. Typically, this value is correspondent with public health guidelines so it has strong applied relevance [14]. Daily dog walking shares this strong applied relevance because it represents a criterion that is meaningful to both human (i.e., 140+ min of walking) and canine (at least daily walking 20+ min) physical activity recommendations [9, 41]. However, given the dichotomization procedure for walking that is used in the creation of the action control framework, it is possible that some of the participants will be classified as unsuccessful intenders from a very minor lapse in walking behavior that isn’t particularly meaningful and this may confound the prediction results and the relevance of the findings. For example, walking six days per week when intending to walk daily represents only a 14% intention-behavior gap. To address the possibility that a minor deviation in behavior is accounting for our findings, we conducted three sensitivity analyses. First, we computed descriptives for the MVPA walking frequency variable among unsuccessful intenders in order to ascertain a basic understanding of the distribution of this group. The group mean and standard deviation should be at five or less bouts of walking in order to show that the dichotomization is meaningful in terms of deviation from daily walking intentions. Second, we recoded those who report six days or more of walking as “successful intenders” and examined whether the proportional shift was significant compared to the original seven day coding. Finally, we re-ran the discriminant function analyses to examine whether any of the findings changed as a result of this recode. The results should not change unless the findings are sensitive to this minor recode. All data are available from the second author.

## Results

### Participant characteristics

A total of 228 respondents began the online survey, but only 227 completed the M-PAC items (see Table 1). The mean age of respondents was 43.11 (*SD* 12.37) years, 88.4% were females, 98.5% were Caucasians, 53.3% were currently employed full-time, 54.2% had four-year college education and above, and 36.5% reported annual household incomes above $100,000. The sample was comparable to census data, with the exception of the inequitable gender response [45]. All participants responded that their dogs were healthy and thus capable of daily walking.

**Table 1:** Sample demographics

Characteristics	
Dog Owner Demographic Profile	
Age in Years (SD)	43.11 (12.37)
% Female	88.4
% Caucasian	98.5
% 4 year college and above	54.2
% Income $100 k and above	36.5
% Full-time Employed	53.3
% Retired	11.0
% Presence of a Yard	92.2
Health Profile	
% Smoker	7.1
Self-Reported Health:	
% Poor	2.4
% Fair	9.9
% Good	36.8
% Very Good	37.7
% Excellent	13.2
Mean BMI (SD)	25.59 (5.04)
Dog Profile	
% Healthy Dogs	100
% Female	50.2
% Small	27.3
% Medium	34.8
% Large	35.7
% Giant	2.2

### Predictors of dog walking action control

The intention-behavior profiles of the action control framework yielded the following distributions: a) non-intenders (26%; *n* = 59), b) non-intenders who did walk daily (1%; *n* = 2), c) unsuccessful intenders (33%; *n* = 75), and d) successful intenders (40%; *n* = 91). Given the small and severely unequal sample size of the non-intenders who walked daily, it was dropped from subsequent analyses as it did not meet power analyses requirements. Overall, the intention-behavior gap (unsuccessful intenders *n* = 73/total intenders *n* = 164) was 45%.

Our preliminary covariate analyses showed that participant demographics (age, gender, income, occupational status, education) and the presence of a residential back yard space were not associated with these action control categories (all *p* > 0.25). Dog breed size, however, was significantly different across the action control framework [*χ* ^2^ (4) = 13.18; *p* < .01), with small breeds more likely to be present in the non-intender and unsuccessful intender categories and larger breed dogs more likely to be present in the successful intender category. Thus, dog breed size was carried forward to the main analyses. The main discriminant analysis identified one discriminant function that significantly distinguished among the three groups [*χ* ^2^ (18) = 75.31, *p* < 0.01; canonical *r* = 0.49]. Affective judgements (*r* = 0.33), automaticity (*r* = 0.38), behavioral regulation (*r* = 0.33) identity (*r* = 0.22) and dog breed size (*r* = 0.28) had meaningful correlations with the discriminant function (see Table 2). Follow-up tests showed that affective judgments, automaticity, and behavioral regulation differentiated non-intenders, unsuccessful intenders, and successful intenders with consecutively larger values in each predictor variable (*d* > 0.30). Identity and dog breed size, however, discriminated between unsuccessful and successful intenders (*d* > 0.30), but not between non-intenders and unsuccessful intenders.

**Table 2:** Prediction of daily dog walking intention-behavior profiles using multi-process action control variables and dog size

	Intention-Behavior Profiles			Correlation with Discriminant Function	Univariate Follow-Up F_2,218_	Post Hocs
	Non-intenders (*n* = 59)	Unsuccessful Intenders (*n* = 75)	Successful Intenders (*n* = 91)			
Dog Breed Size	2.05 (0.87)	1.89 (0.86)	2.34 (0.77)	.28	6.33*	NI,UI < SI
Outcome Expectations (Human)	3.98 (0.77)	3.92 (0.83)	4.28 (0.78)	.14	NA	NA
Outcome Expectation (Canine)	4.60 (0.52)	4.65 (0.47)	4.81 (0.40)	.07	NA	NA
Affective Judgements	4.10 (0.82)	4.35 (0.59)	4.69 (0.41)	.33	18.43*	NI < UI < SI
Perceived Capability	4.48 (0.98)	4.73 (0.52)	4.76 (0.61)	-.03	NA	NA
Perceived Opportunity	4.27 (0.84)	4.54 (0.61)	4.62 (0.67)	.03	NA	NA
Behavioral Regulation	2.73 (0.93)	3.04 (0.93)	3.42 (0.92)	.33	10.27*	NI < UI < SI
Automaticity	3.00 (1.02)	3.63 (1.09)	3.96 (0.98)	.38	15.65*	NI < UI < SI
Identity	3.33 (1.01)	3.49 (0.85)	4.07 (0.63)	.22	17.53*	NI,UI < SI

Note: * = *p* < 0.01. *NI* = non-intenders, *UI* = unsuccessful intenders, *SI* = successful intenders. *NA* = not applicable. Post hoc tests interpreted as *p* < 0.05 and *d* > 0.30 based on the recommended minimum effect size for social science data (Ferguson [43]; Cohen [44])

### Sensitivity analysis

The unsuccessful intenders group reported a mean of 2.97 bouts of walking with a standard deviation of 2.36. This is a sizeable deviation from their reporting of intended daily walking, suggesting meaningful intention-behavior discordance in this group. Recoding of the participants who reported six walks as “successful intenders” however, did shift 12 participants (16%) out of the unsuccessful intenders category and this was a significant change to the proportions [Cochran’s Q (1) = 12.00; p < 0.01]. Results from the discriminant function analysis with this new categorization were almost identical to the original action control classification [*χ* ^2^ (18) = 88.16, *p* < 0.01; canonical *r* = 0.49] with the same overall findings and no deviations in the results by p-value or effect size classifications. Thus, the results were not sensitive to minor deviations in intention and behavior.

## Discussion

This study was the first to examine the intention-behavior gap in daily walking behavior among dog owners and predict this gap using the M-PAC framework designed for this purpose [16, 17]. We hypothesized that three of four possible intention-behavior profiles would emerge (i.e., non-intenders, unsuccessful intenders, successful intenders), commensurate with prior research in general PA [14]. This hypothesis was supported. Only two participants in the sample were classified as having low intentions and engaged in daily dog walking. By contrast, 73% of the sample was comprised of intenders, yet 45% of these intenders were not walking congruent with their intentions and most of these participants walked <50% below what they intended.

The finding has both theoretical and applied implications. From a theoretical perspective, the results demonstrate that intention formation is a necessary process but it may be insufficient for walking enactment among many dog owners. Thus, frameworks that propose intention as the bridge to behavior, which comprise many of our most popular health behavior models and contemporary models for dog walking [10], may not be as useful as models that separate intention translation from intention formation [16]. From an applied perspective, these results also help explain that some dog owners have yet to form daily walking intentions, while even more participants intend to walk their dog daily but fail to follow-through. Thus, dog walking promotion may benefit from both intention formation and action control interventions, depending on the readiness of the population.

In light of this aim, the second purpose of the study was to predict these intention-behavior profiles using the M-PAC framework. The findings support almost all of the tenets of that model. M-PAC suggests that instrumental outcome expectations about walking may not be as important to action control because they do not reflect the experience of the action itself. Perceptions of capability are also generalized to the act (i.e., am I physically able to walk or not) and not specific to each action but could be important to dog owners given the additional demands of controlling dog behavior. Congruent with theory, outcome expectations and perceived capability to walk did not contribute to the intention-behavior profiles while controlling for other M-PAC variables. Thus, educational/informational campaigns based on the benefits of walking and interventions to improve one’s ability to walk are not recommended as standalone interventions for closing the intention-behavior gap, even though these constructs have been shown to be general correlates of owner dog walking [11].

By contrast, affective judgements did contribute to the discriminant function, predicting all three intention-behavior profiles. While affective judgments have been shown to predict owner intentions to dog walk in prior research [13], this finding supports the approach taken in M-PAC [17], where higher affect is also needed to enact a behavior than form the intention. From a theoretical perspective, the importance of affective over instrumental outcome expectations during action control supports hedonic theories of behavior. The practical aspect of this finding suggests that consideration of pleasure in walking interventions may facilitate closing the intention-behavior gap. M-PAC constructs are considered the consequence of individual, social, and environmental/policy factors [46]. For affective judgments, this could involve several factors that require future research, such as pleasant walking conditions (e.g., environmental design and dog-friendly amenities), social aspects of walking (e.g., walks with friends/ other dog owners), and dog-related variables that could affect the walking experience (e.g., level of training and responsiveness, dog’s sociability towards other dogs and people, owner enjoyment related to bonding with their dog).

The hallmark of most action control models, including M-PAC, is the premise that volitional self-regulation tactics are needed to tie good intentions to behavior [16]. In support of this hypothesis, behavioral regulation correlated with the discriminant function and predicted all three intention-behavior profiles. The inclusion of behavioral regulation variables in understanding general PA has had considerable support [47] but this is the first study in dog owners to apply this variable to our knowledge. The results suggest that having dog owners make action (when, where, how, and with whom) and coping (details about how to overcome potential set-backs) plans and subsequently track these plans (e.g., with mobile phone apps, diaries) may be very useful to close the intention-behavior gap.

The reflexive M-PAC constructs of habit/automaticity and identity also contributed to the discriminant function. Habit predicted all three intention-behavior profiles while identity was considerably higher for successful intenders compared to unsuccessful intenders. These are new constructs to the dog owner and walking correlates literature, and represents potentially important considerations for future intervention. Both constructs are considered for the maintenance of action control, as performance experience with the behavior is necessary for their formation. From a theoretical standpoint, habits are thought to be formed from consistent repetitions of action and exposure to similar cues [23], which highlight the importance of environmental and social context. Preliminary research suggest that the key to habit formation is consistency of practice [48]. Dog owners with a regular walk routine may thus be more likely to acquire automaticity over time and facilitate action control partially independent of motivation. Given the long period of owning a dog, this may be a critical factor to behavior maintenance over time.

Our measure of dog walking identity in this study may overlap with prior research on dog support/obligation (e.g., [12, 13, 49]). Identity is a self-categorization of oneself into a particular role [25] and a sense of obligation and responsibility for one’s dog would seemingly be a part of a dog walking identity. Our findings with identity and action control support this prior work. Qualitative interviews with dog owners highlight how dog walking is a duty or a role described with similarities to parenting children [50]. Further, Brown and Rhodes [13] found that a sense of dog responsibility/obligation was able to predict walking behavior independent of walking intention, which is similar to the results of the current study. In M-PAC, identity is expected to impact action control via selective processing of information, thus shielding from other intentions (i.e., staying on course) and by imposing dissonance (i.e., negative affect) when behavior is not congruent with the identity [16]. It seems worthy to explore whether dog walking identity can be modified, presumably through pre-set rank-ordering (e.g., priority lists, I will walk the dog before I do household chores), commitment affirmations (realizations and statements about the value of dog walking to companionship), and social activations (purposeful statements about dog walking when describing one’s self to others) [51].

The only variable discrepant with our hypotheses was perceived opportunity, which did not contribute to the discriminant function with a meaningful effect size. Our assessment of this construct used Williams and Rhodes’ [37] suggestion to include a motivational qualifier in the assessment (i.e., if I wanted to), which may have explained the null result as opportunity was circumscribed from motivation. Westgarth and colleagues [10] point out that opportunities to dog walk is not a consistent correlate within the dog walking literature, and it may also not be a critical predictor of action control.

Interestingly, dog breed size also predicted action control independent of the M-PAC variables. Those owners with larger dogs were more likely to enact their walking intentions compared to those with smaller dogs, yet dog size did not distinguish between non-intenders and unsuccessful intenders. The finding is interesting and may highlight how the dog influences walking independent of initial owner-related walking motivations, although assessment with a stronger design (i.e., longitudinal, experimental) is needed to advance this conjecture. Large dogs have higher energy expenditure needs than smaller dogs [9] so this finding may represent the dog’s influence on action control. Regular dog walking has been characterised as a unique physical activity due to the symbiotic inter-dependency between the canine and owner [26] and dog characteristics have been correlated with regular walking [10]. This is the first study to examine action control of dog walking, but future research into the role the dog plays in facilitating or inhibiting owner intentions seems warranted.

Despite the novel findings of this study, the results need to be considered within the context of its limitations and these prompt areas for future research. First, the survey was cross-sectional, making interpretations limited to the assumption that past walking is a good predictor of future dog walking. Second, the assessment of walking is subject to self-report bias. It would stand to reason that an objective assessment of walking would be ideal and a more stringent test of the M-PAC model. Third, the outcome expectation, perceived capability, and perceived opportunity items had limited items that might be compromising the reliability and validity of these constructs. Replication with measures employing multi-item measures with test-retest validation, frames other than daily walking (e.g., 5 times per week) and more dog-related and environmental characteristics is warranted. Finally, the sampling frame was limited to those who visit dog-related social media, posters, and advertisements around Victoria, Canada, and included mainly affluent middle-aged non-Hispanic white women. Recent research suggests that the effects of dog ownership on walking may not extend across racial/ethnic groups [6], so a broader sampling approach is needed in future research in order to examine whether these findings replicate to other regions and participants with different socio-demographic profiles.

## Conclusions

Overall, the findings demonstrate that dog walkers show an intention-behavior profile similar to general physical activity research, with almost half of those intending to engage in daily walks falling short of this criterion. Using the M-PAC framework, differences between successful and unsuccessful intenders were associated with affective judgments about walking, behavioral regulation tactics, habit, and identity. Addressing these should be the focus of interventions aimed at improving dog walking.

## Appendix

**Table 3:** Predictor measures where regular dog walking was defined as daily walking at a moderate or higher intensity

Construct	Items	Original Source (s)	Reliability
Automaticity	1) I engage in *dog walking* automatically 2) I engage in *dog walking* without having to consciously remember it 3) I engage in *dog walking* without consciously thinking about it	Gardner et al. [39]; Verplanken & Orbell, [52]	α = .93
Behavioral Regulation	1) I made detailed plans regarding what to do if something interfered with my plans to engage in *dog walking* over the past week 2) I reserved time in my daily schedule for regular *dog walking* over the past week 3) I made plans concerning “when”, “where”, “what” and “how” I was going to engage in *regular dog walking* over the past week	Sniehotta et al. [38]	α = .76
Identity	1) I consider myself someone who is physically *active with my dog* 2) When I describe myself to others, I usually include being *physically active with my dog* 3) Others see me as someone who *walks my dog regularly*	Anderson & Cychosz, [53]; Wilson and Muon [40]	α = .87
Affective Judgments	1) I *walk my dog* because it is fun 2) I enjoy *my dog walking* sessions 3) I find *dog walking* a pleasurable activity 4) I get pleasure and satisfaction from engaging in *dog walking*	Markland and Tobin [35]	α = .92
Perceived Capability	1) I am physically able to *walk my dog regularly* if I wanted to	Rhodes et al. [36]; Williams & Rhodes [37]	
Perceived Opportunity	1) I have the opportunity to *walk my dog regularly*if I wanted to	Rhodes et al. [36]	
Dog Outcome Expectation	1) *Regular dog walking* would help keep my dog healthy	Cutt et al [34]	
Human Outcome Expectations	1) *Regular dog walking* would help me to maintain or lose weight 2) Regular dog walking would allow me to get to know my neighbourhood	Cutt et al [34]	α = .69
Intention	Over the next week, I intend to walk the dog ___________ days per week.	Courneya [33]	

## Abbreviations

M-PAC: Multi-Process Action Control
